# Incidence and remission of aeroallergen sensitization in adults in Northern Finland: 15 years longitudinal study

**DOI:** 10.1038/s41598-021-83326-6

**Published:** 2021-02-19

**Authors:** Anna Karoliina Haarala, Suvi-Päivikki Sinikumpu, Eeva Vaaramo, Jari Jokelainen, Markku Timonen, Juha Auvinen, Juha Pekkanen, Jussi Lampi, Laura Huilaja

**Affiliations:** 1 PEDEGO Research Unit, PEDEGO Research Group, Department of Dermatology, University Hospital of Oulu and Medical Research Center, University of Oulu, Aapistie 5A, 90029 Oulu, Finland; 2 Infrastructure for Population Studies, Faculty of Medicine, University of Oulu, Oulu, Finland; 3 Center for Life Course Health Research, Faculty of Medicine, University of Oulu, Oulu, Finland; 4 Medical Research Center, Oulu University Hospital, Oulu, Finland; 5 Department of Public Health, University of Helsinki, Helsinki, Finland; 6 Finnish Institute for Health and Welfare, Helsinki, Finland; 7 Department of Health Security, Environmental Health, Finnish Institute for Health and Welfare, Kuopio, Finland

**Keywords:** Skin diseases, Epidemiology, Diseases, Medical research

## Abstract

Studies on the longitudinal changes in sensitization to aeroallergens in adult populations are sparse. The aim was to evaluate changes in sensitization to aeroallergens [birch, timothy, cat and house dust mite (HDM)] in an unselected adult population aged from 31 to 46 years. Data were gathered from a cohort of adults (Northern Finland Birth Cohort 1966) who had been skin prick tested (SPT) with birch, timothy, cat and HDM allergens at the age of 31 years and at age 46 (n = 5484 and 5373 respectively). Data from both time points were available for 3409 participants, who made up the cohort of the longitudinal study. The overall prevalence of sensitization to any of the selected allergens was 30.3% (n = 1661) in 31-year-olds and 30.7% (n = 1649) in 46-year-olds. In general, men were more sensitized (*P* < 0.001) and also had more polysensitization (*P* < 0.001) compared to women. In longitudinal sub-population incidence of sensitization was 7.1%. Birch was the most prevalent new sensitizer, however, the difference was not statistically significant when compared to cat. We conclude that new sensitization, demonstrated by positive findings in SPT, can still occur in middle age and this should be taken into account when managing allergic manifestations in adults as sensitization can be considered the first step in developing clinical allergy.

## Introduction

The high prevalence of allergic diseases is a major health concern worldwide^1^. It has also been shown that until mid-adulthood most cases of asthma are related to other allegic diseases^2^ and allergic comorbidities are related to the severity of astma^3^. Allergic diseases substantially reduce quality of life and, especially if treated inadequately, cause a burden both to the individual and to society in the form of healthcare costs and impaired work performance^4^.

Cross-sectional studies report that allergic sensitization is very common in the general adult population: a study conducted in Helsinki, Finland (n = 498; 26–60 years) found that nearly half of the adult population (46.9%) tested for 15 aeroallergens had at least one positive skin prick test (SPT)^5^. Similarly, almost 40% of adult subjects (20–44 years) were found to be sensitized in an Estonian study (n = 516)^6^. In a U.S. study of allergies carried out in 2005–2006 in individuals aged 30–59, around 30% of the study population of this age group were tested positive for at least one aeroallergen immunoglobulin E (IgE)^7^. More recently, a Swedish study (n = 802) found allegic sensitization in 44.6% of adults (31–45 years) when defined as at least one positive SPT^8^.

Previous longitudinal studies have reported that sensitization to aeroallergens increases with age at least until early adulthood^9–13^. In Denmark, a study of repeated specific IgE (sIgE) assessments performed during the years 1985–2011 found an increase in sensitization to aeroallergens when participants were followed from the age of 1–26 years^10^. In the U.S. in 1987, a cohort study of 1333 subjects (age ≥ 3 years), who were followed 6–10 years, found that the prevalence of sensitization peaked between the ages of 25 and 34 years (53.5%)^11^. In an Italian longitudinal study of 788 participants, sensitization to common allergens increased with age, but mainly in subjects under the age of 40 years^12^. In a multicenter study, in which 3206 European and Australian adults were followed over 20 years, sensitization especially to cat and house dust mite (HDM) was found to decrease after 20 years of age^14^. Currently, there are only limited and inconsistent data on changes in sensitization in adulthood^14–16^. It is well described that the prevalence of allegic sensitization increases by increasing age from childhood to early adulthood starting to decrerase thereafter^9,10,14,17^. However, since the prevalence of sensitization is a result of both incidence and remission, longitudinal setting is needed to analyse changes in sensitization in adults. Thus, the aim of this study was to investigate incidence, remission and longitudinal changes in prevalence of sensitization in a large adult cohort in Northern Finland followed from age 31 to 46 years. Secondary, we aimed to analyze the prevalence of polysensitization to common aeroallergens and to study differences in sensitization between genders among unselected adult population.

## Results

Baseline characteristics of the study population are described in Table 1.

**Table 1:** Characteristics of the study populations.

	31 years study N (%) (‘Cohort 31’)	46 years study N (%) (‘Cohort 46’)	Longitudinal study^a^ N (%) (‘Longitudinal sub-population’)
Total	5484	5373	3409
Men	2733 (49.8)	2394 (44.6)	1572 (46.1)
Women	2751 (50.2)	2979 (55.4)	1837 (53.9)

^a^Sub-population of those participants who had non missing SPT data on both time points (31 and 46 years).

### Incidence and remission of allergic sensitization between 31 and 46 years

Participants who had non-missing SPT data on all allergens at both time points (n = 3409) were included in the longitudinal analysis; of these, 1837 (53.9%) were female. During the 15-year new sensitization (among those without any positive reaction at the age of 31 years but who became sensitized for at least one allergen, during the follow-up period) occurred in 7.1% of participants (Table 2). New sensitization occurred more often in women than in men (7.7% vs. 6.5%) but this finding was not statistically significant (Table 3). New sensitization occurred in 4.6% for birch, 4.3% for cat, 3.3% for timothy and 2.4% for HDM (Table 2): the difference between birch and other allergens was statistically signifigant except for cat (*P* = 0.417, data not shown). In addition, 6.0% of women and 5.5% of men who had been sensitized to at least one aeroallergen at the age of 31 lost their sensitization completely during the follow-up period. In general, men had higher risk to stay sensitized from 31 to 46 years when compared to women (*P* < 0.001) (Table 3). During the follow-up period, 66.1% of women and 61.7% of men stayed non-sensitized (no positive prick test reactions at either the age of 31 or 46 years) (Table 3).

**Table 2:** Consistency of sensitization between ages 31 and 46 by allergen.

SPT Consistency		Cat		Birch		Timothy		HDM		All	
31 years	46 years	n	%	n	%	n	%	n	%	n	%
N	P	145	4.3	158	4.6	112	3.3	82	2.4	243	7.1
P	N	147	4.3	142	4.2	109	3.2	133	3.9	196	5.8
P	P	374	11.0	390	11.4	399	11.7	76	2.2	787	23.1
N	N	2743	80.5	2719	79.8	2789	81.8	3118	91.5	2183	64.0
All	All	3409	100.0	3409	100.0	3409	100.0	3409	100.0	3409	100.0

*SPT* skin prick test, *HDM* house dust mite, *N* negative, *P* positive.

**Table 3:** Consistency of SPT findings between ages 31 and 46 by sex.

31 years	46 years	Cat			Birch			Timothy			HDM			All		
		F	M	P*	F	M	P*	F	M	P*	F	M	P *	F	M	P*
		n (%)	n (%)		n (%)	n (%)		n (%)	n (%)		n (%)	n (%)		n (%)	n (%)	
**SPT consistency**				0.018			0.152			< 0.001			0.120			< 0.001
N	P	81 (4.4)	64 (4.1)	0.636	93 (5.1)	65 (4.1)	0.204	54 (2.9)	58 (3.7)	0.216	46 (2.5)	36 (2.3)	0.692	141 (7.7)	102 (6.5)	0.186
P	N	81 (4.4)	66 (4.2)	0.774	79 (4.3)	63 (4.0)	0.680	60 (3.3)	49 (3.1)	0.815	69 (3.8)	64 (4.1)	0.653	110 (6.0)	86 (5.5)	0.529
P	P	173 (9.4)	201 (12.8)	0.002	192 (10.4)	198 (12.6)	0.047	175 (9.5)	224 (14.3)	< 0.001	31 (1.7)	45 (2.9)	0.020	373 (20.3)	414 (26.4)	< 0.001
N	N	1504 (81.8)	1239 (78.9)	0.035	1475 (80.2)	1244 (79.2)	0.482	1550 (84.3)	1239 (78.9)	< 0.001	1693 (92.1)	1425 (90.8)	0.177	1215 (66.1)	968 (61.7)	0.008
All	All	1839 (100)	1570 (100)		1839 (100)	1570 (100)		1839 (100)	1570 (100)		1839 (100)	1570 (100)		1839 (100)	1570 (100)	

*HDM* house dust mite, *SPT* skin prick test, *F* female, *M* male, *y* years, *N* negative, *P* positive, *Ref* Reference.

*P value for difference between sexes.

### Prevalence at age 31 and 46 years

Tables 4 and 5 show the overall prevalence of sensitization both in Cohort-31 and Cohort-46 populations (n = 5487 and n = 5373, respectively) (Table 4) and in a longitudinal sub-population (n = 3409, Table 5). The overall prevalence of sensitization remained stable in cohorts being 30.3% (n = 1661) at the age of 31 and 30.7% (n = 1649) at the age of 46. Corresponding prevalences for sensitization were seen in longitudinal sub-population (28.8% and 30.2%, respectively).

**Table 4:** Prevalence of sensitization at 31-year and 46-year studies by sex.

	31 years				46 years			
	All	M	F	P*	All	M	F	P*
n	5484	2733	2751		5373	2394	2979	
Sensitization	1661 (30.3%)	904 (33.1%)	757 (27.5%)	< 0.001	1649 (30.7%)	794 (33.2%)	855 (28.7%)	< 0.001
Cat	876 (16.0%)	464 (17.0%)	412 (15.0%)	0.047	829 (15.4%)	394 (16.5%)	435 (14.6%)	0.067
Birch	889 (16.2%)	478 (17.5%)	411 (14.9%)	0.012	907 (16.9%)	421 (17.6%)	486 (16.3%)	0.230
Timothy	870 (15.9%)	489 (17.9%)	381 (13.8%)	< 0.001	852 (15.9%)	450 (18.8%)	402 (13.5%)	< 0.001
HDM	366 (6.7%)	214 (7.8%)	152 (5.5%)	0.001	257 (4.9%)	126 (5.3%)	131 (4.4%)	0.157

*HDM* house dust mite.

*P value for difference between sexes.

**Table 5:** Prevalence of sensitization at age 31 and 46 in longitudinal sub-population by sex.

	31 years				46 years			
	All	M	F	P*	All	M	F	P*
n	3409	1570	1839		3409	1570	1839	
Sensitization	983 (28.8%)	500 (31.8%)	483 (26.3%)	< 0.001	1030 (30.2%)	516 (32.9%)	514 (27.9%)	0.002
Cat	521 (15.3%)	267 (17.0%)	254 (13.8%)	0.011	519 (15.2%)	265 (16.9%)	254 (13.8%)	0.015
Birch	532 (15.6%)	261 (16.6%)	271 (14.7%)	0.142	548 (16.1%)	263 (16.8%)	285 (15.5%)	0.344
Timothy	508 (14.9%)	273 (17.4%)	235 (12.8%)	< 0.001	511 (15.0%)	282 (18.0%)	229 (12.5%)	< 0.001
HDM	209 (6.1%)	109 (6.9%)	100 (5.4%)	0.079	158 (4.6%)	81 (5.2%)	77 (4.2%)	0.206

*HDM* house dust mite.

*P value for difference between sexes.

**Figure 1:**
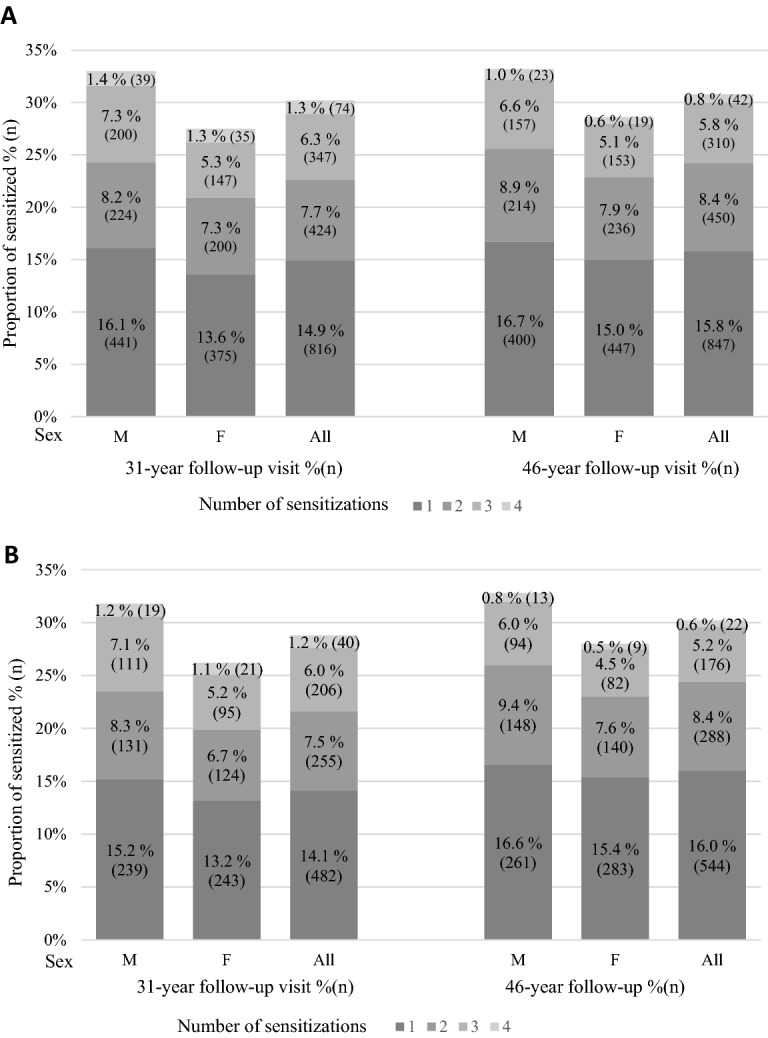
Prevalence of sensitization and polysensitization by sex at the 31- and 46-year follow-up visit. (**A**) All study subjects, (**B**) Longitudinal sub-population. *P* < 0.001 for polysensitization between sexes in both settings in both time points. Shades of gray representing amounts of sensitizations : (1) one sensitization, (2) two sensitizations, (3) three sensitizations, (4) four sensitizations.

Polysensitization (two or more positive reactions to SPT) occurred in 845 (15.4%) of participants at age 31 and in 802 (14.9%) at age 46 and in 501 (14.7%) and 486 participants in (14.2%) in longitudinal sub-population, respectively (Fig. 1). Polysensitization was significantly more frequent in men than in women at both time points in both Cohort 31 and Cohort 46 (Fig. 1A) as well as in longitudinal sub-population (*P* < 0.001) (Fig. 1B).

Overall sensitization was more common in men than in women in both time points (*P* < 0.001). In total cohort, at the age of 31 this difference was statistically significant for all allergens (*P* < 0.05). The difference between sexes at the age of 31 was most evident for timothy (*P* < 0.001), which became even more apparent at the age of 46. Timothy was the only allergen to retain a statistically significant sex difference at age 46 (*P* < 0.001). The prevalence of sensitization by sex is shown in Table 4.

## Discussion

In this unique and expectionally large longitudinal study among unselected adult population we present that as many as 7.1% of adults became sensitized. Only a few previous longitudinal studies have reported on sensitization to aeroallergens in adult populations^12,14–16^. Some of these studies have reported an increase over time in sensitization in this age group, while others have found a decrease. For example, a multicenter European study with over 20 years follow-up found that the prevalence of sensitization to cat, HDM and grass and total IgE decreased especially among those aged 48 years or older^14^. A study conducted in Sweden (N = 664) reported a low incidence and high remission of allergic sensitization in a 10 years period^16^. However, over 50% of the participants were older than 50 years already at the beginning of the study. In a Danish study based on the data from 1970s, they found 7% cumulative incidence of allergic sensitization between the ages of 40 and 60^15^. Correspondingly, the longitudinal analysis of our study found that new sensitization occurred in 7.1% of participants. A more recent Korean study (n = 138) reported a slightly higher rates (10.5%) of new sensitization among 40–59 years old patients (n = 35) with rhinitis^17^. Interestingly, our results were comparable to those from the Danish study although they had a remarkably smaller study population (n = 695), but the follow up period was 5 years longer than in our study^15^. Varying age groups and follow-up times make direct comparisons difficult since longer observation time allows higher probability of new events.

We found that between 31 and 46 years the prevalence of sensitization remained quite stable (30.3–30.7%) due to similar numbers of those who lost sensitization and those who developed sensitization. This is in line with the findings from previous cross-sectional studies^12,18,19^, except a Swedish study which reported a prevalence of sensitization in 31–45 years olds being 44.6%^8^. In the longitudinal follow-up, the most common new sensitizer was birch although this was not statistically significant when compared to cat. Correspondingly, birch was also the most prevalent sensitizing allergen at both time points (in total approximately 17% of the population were affected at each time point). Other Northern European studies have also reported birch to be an important allergen^18,20,21^. During the follow-up period 5.8% lost sensitization completely, commonly for cat. This finding may result from diminished contacts with cats as they are not so popular as pets as the dogs are^22^. It is also reported that the relation between exposure and sensitization is less well defined to cat than for example to pollens^23^.

In the present study, both at age 31 and 46 years, sensitization to timothy grass was found in around 15% of the population, which can be considered moderately high, although studies with cohorts younger than ours have found sensitization to grass to be even more common^14^. The decrease in HDM sensitization (from 6.7 to 4.9%) probably reflects the fact that only minimal amounts of HDMs or their allergens are found in Finnish homes^24,25^. Although HDM sensitization is increasing worldwide simultaneously with urbanization^26^, it is possible that extreme climate conditions and a constant need for central heating keeps the relative humidity levels low and thus unfavorable to especially to *D. pteronyssinus*^26^. Our study shows that HDM has only little importance as an aeroallergen in Northern Finland.

Polysensitization has increased during last decades^18,19^ and it is considered especially important as it has been linked to asthma in adults^27^ and allergic multimorbidity in both children and adults^28–30^. Polysensitization was common in our study, with around 15% of the participants at both time points having two or more sensitizations. Even higher polysensitization (defined as ≥ 3/10 positive SPT) rates of up to 56% were found in Swedish study^18^. The fact that the higher the number of allegens tested the higher the probability for positive findings may partly explain the high polysensitization rate found in their study.

In the present study, males were more sensitized and also had more polysensitization. Sex differences in rates of sensitization have been reported before^7,31–33^. A study from UK reported that boys under 18-years-old are significantly more likely to be sensitized than girls^9^. However, in Swedish study the effect of gender decreased by adolescence^13^. In a large national US health study including both children and adults, overall sensitization was more common in males^7^. This has been explained by genetic differences between sexes that manifest from infancy^33^, but the exact factors are unknown. Surprisingly, in our longitudinal sub-population, females were slightly more prone to develop a new sensitization between the ages of 31 and 46.

The strengths of the study include its large, unselected population and the longitudinal follow-up data. The relatively high participation rate allows our results to be generalized to the entire population. We used the SPT technique, which is considered the gold standard for detecting allergic sensitization^34^ and has a good positive predictive value to determine clinical allergy in respiratory allergic diseases^35^. The similar prevalence estimates at age 31 and 46 years in the different samples (the two cross-sectional and the longitudinal sample) further support the representability of the longitudinal results (incidence and remission). The lack of concurrent sIgE assessment can be considered as a limitation of this study. Some participants declined the SPT for unknown reasons. Consequently, some of the most sensitized individuals might not have been included in the analyzed population. Furthermore, there was a possibility of selection bias in the population who participated in the clinical examinations. The permitted use of antihistamine medication may have affected the outcome of the SPT, but despite this possibility, these results indicate that sensitization is very common in the adult population of Northern Finland. Allergens differ by geographical area^36^, and this affects the findings of studies on allergic sensitization. In addition, the fact that this study was performed in a high-income country may limit its generalizability to other populations^37^.

In conclusion, this study adds important information that sensitization does not decrease before 46 years, which makes it an important health factor throughout middle age. Rather, the present study showed that new onset sensitization, demonstrated by positive findings in SPT, can still occur in middle age and this should be taken into account when managing allergic manifestations in adults.

## Methods

### Study population

**Figure 2:**
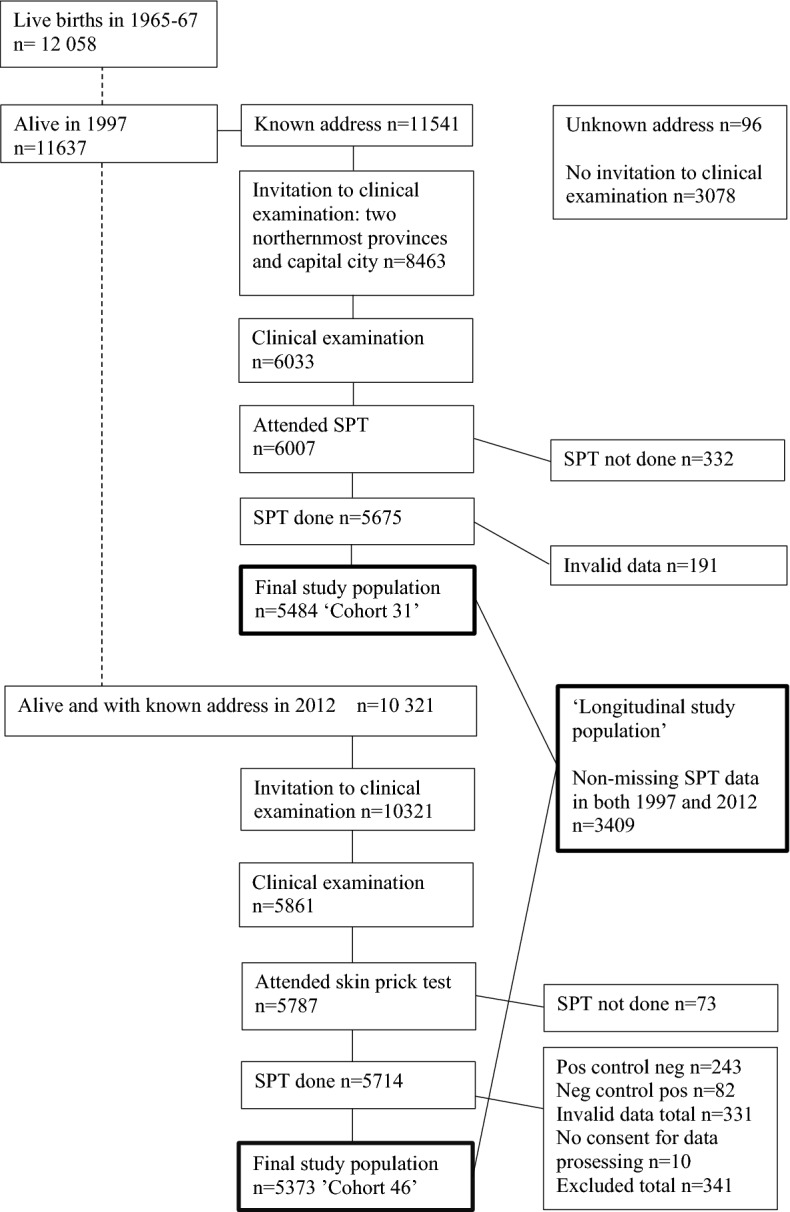
Flow chart of the NFBC 1966 population: follow-up visits including skin prick tests (SPT).

Our study data were drawn from the Northern Finland Birth Cohort 1966 (NFBC 1966) which is a longitudinal research program in the two northernmost provinces in Finland. The NFBC 1966 initially included all 12,058 children whose expected date of birth fell in the year 1966. The whole cohort has been evaluated regularly since birth by way of health questionnaires and clinical examinations including skin examination^38,39^. To date, the cohort has been subject to four main follow-up visits: at birth, and at the ages of 14, 31 and 46 years. SPTs were performed at the ages of 31 and 46 (Fig. 2). Detailed information on the NFBC 1966, including flow charts of the 31- and 46-year follow-up visits can be seen on the research program’s website^40^.

At 31 years, invitations to the clinical examination were sent to 8463 cohort members and 6033 participated. At 46 years, invitations were sent to every living member of the cohort whose address was known (n = 10,321) and 5861 participants attended the clinical examination day. In the 31- and 46-year follow-up studies SPTs were performed to 5675 (67.1% of invited) and 5714 (55.4% of invited) participants, respectively (Fig. 2). Because of invalid data, 191 and 331 participants were excluded from the 31- and 46-year follow-up analyses, respectively. The final study population consisted of participants who had non-missing SPT data on all allergens: 5484 study subjects at the age of 31 (‘Cohort 31’) and 5373 at the age of 46 (‘Cohort 46’). The same participants, who had non-missing SPT data on all allergens at both time points (n = 3409), were included in the longitudinal analysis (‘longitudinal sub-population’) (Fig. 2).

### Skin prick test

The present study analyzed available data from SPTs. SPTs were conducted with standard dilutions of three of the most common allergens in Finland (cat, birch, and timothy), plus HDM (Dermatophagoides pteronyssinus) (Alk-Abello Nordic, Espoo, Finland). Histamine dihydrochloride (10 mg/ml) and diluent of the allergen extracts were used as positive and negative controls, respectively. SPTs were performed on the volar surface of the forearm. After 15 min, the skin reactions to each allergen were recorded as the average of the maximum wheal diameter and the diameter perpendicular to the maximum value. A wheal ≥ 3 mm was considered positive and was counted as confirmation of sensitization for study purposes^34^. Tests were performed by trained nurses and both cross- and double-checked regularly. Because of the study design, all regular medications were permitted, including antihistamines. There were N = 74 antihistamine users in 31 years study and N = 367 in 46 years follow-up study. Subjects with a negative histamine reaction (wheal size < 3 mm) and subjects with a positive reaction to the negative control were excluded from the analysis. A new variable was created to allow analysis of sensitization status during the follow-up period. The variable was dichotomized as follows: sensitized at neither time point, or sensitized at one or both time points.

### Ethics statement

The Ethical Committee of the Northern Ostrobothnia Hospital District approved the study, which was performed according to the Helsinki Declaration of 1983. Written informed consent for scientific purposes was received from all participants.

### Statistical analysis

Cross-tabulation was used to analyze associations of sex with overall sensitization, polysensitization, and sensitization to the individual allergens, which were presented as frequencies and percentages. The Chi-squared test was used to test differences between sexes. McNemar's test was used to analyze changes in overall sensitization and sensitization to birch, timothy, cat and HDM by sex and also when analyzing differences between allergens. *P* values of < 0.05 were considered statistically significant. The analyses were conducted using the SAS software package (version 9.4, SAS Institute Inc., Cary, NC, USA) and R version 3.1.2 (https://cran.rstudio.com).

## Data Availability

The data that support the findings of this study are available from Northern Finland Birth Cohort 1966 Study. Restrictions apply to the availability of these data, which were used under license for this study. Data are available at http://www.oulu.fi/nfbc/node/44315 with the permission of Northern Finland Birth Cohort.

## Abbreviations

NFBC 1966: Northern Finland Birth Cohort 1966
IgE: Immunoglobulin E
SPT: Skin prick test
HDM: House dust mite
